# Subtyping the Autism Spectrum Disorder: Comparison of Children with High Functioning Autism and Asperger Syndrome

**DOI:** 10.1007/s10803-018-3689-4

**Published:** 2018-07-24

**Authors:** Concetta de Giambattista, Patrizia Ventura, Paolo Trerotoli, Mariella Margari, Roberto Palumbi, Lucia Margari

**Affiliations:** 1 Child Neuropsychiatry Unit, Department of Basic Medical Sciences, Neuroscience and Sense Organs, Hospital Polyclinic of Bari, University of “Aldo Moro” Bari, Piazza Giulio Cesare 1, 70100 Bari, Italy; 2 Medical Statistic, Department of Biomedical Science and Human Oncology, University of “Aldo Moro” Bari, Bari, Italy

**Keywords:** Asperger syndrome, High functioning autism, Autism spectrum disorders, DSM-5, Subtyping

## Abstract

Since Hans Asperger’s first description (Arch Psych Nervenkrankh 117:76–136, 1944), through Lorna Wing’s translation and definition (Psychol Med 11:115–129, 1981), to its introduction in the fourth edition of the Diagnostic and Statistical Manual of Mental Disorders (DSM, 1994), Asperger Syndrome has always aroused huge interest and debate, until vanishing in the DSM fifth edition (2013). The debate regarded its diagnostic validity and its differentiation from high functioning autism (HFA). The present study aimed to examine whether AS differed from HFA in clinical profiles and to analyze the impact of DSM-5’s innovation. Differences in cognitive, language, school functioning and comorbidities, were revealed when 80 AS and 70 HFA patients (3–18 years) were compared. Results suggested that an AS empirical distinction within autism spectrum disorder should be clinically useful.

## Introduction

### A Concise History of Asperger Syndrome

In the same years of Leo Kanner’s description of Infantile Autism (Kanner 1943), Hans Asperger wrote a case report on Autistic Psychopathy (Asperger 1944), describing children with social-communication impairment, eccentric manners, unusual interests and cognitive domains of hyper-functioning. The American Psychiatric Association (APA) did not immediately recognize Autism as a distinct category: it was introduced as Infantile Autism in the third edition of the Diagnostic and Statistical Manual of Mental Disorders (DSM-III, APA 1980) and included within Pervasive Developmental Disorders (PDD) in the DSM-III-R (APA 1987). In 1981, Wing (1981) resumed Asperger’s researches, renaming the Autistic Psychopathy as Asperger Syndrome (AS). In 1989, the first diagnostic criteria for AS were proposed (Gillberg and Gillberg 1989; Szatmari et al. 1989) and in the 1990s, AS appeared in the DSM-IV within PDD (APA 1994).

### Asperger Syndrome and High Functioning Autism

The diagnosis of AS required at least two symptoms of social interaction impairment and one symptom of behavioral and interest restriction, a normal cognitive functioning and the absence of significant general delay in language. Moreover, diagnostic criteria for Autistic Disorder should not be met (otherwise, Autistic diagnosis should have precedence). This implied a differential diagnosis between AS and Autism, especially the type without cognitive delay, also known as High Functioning Autism (HFA) (Klin et al. 2000, 2005). HFA is not a term used in the DSM, but it is commonly used to identify patients diagnosed with Autistic Disorder (AD) or PDD-Not Other Specified (PDD-NOS), with average or above average intellectual abilities (Intelligent Quotient, IQ, higher than 70). HFA differs from low-functioning autism (IQ lower than 70) in terms of clinical presentation, prognosis and need of support and assistance in daily life. Since AS and HFA are both characterized by a normal cognitive functioning, there has been considerable debate over whether AS and HFA are distinct conditions, suggesting different etiological and neurobiological mechanism, or share a similar underlying neuropsychological functioning and should therefore be regarded as variants of a single disorder (Gilchrist et al. 2001; Howlin 2003; Ghaziuddin and Mountain-Kimchi 2004; Macintosh and Dissanayake 2004; Walker et al. 2004; Koyama et al. 2007; Saulnier and Kin 2007; Bennett et al. 2008; Ghaziuddin 2008; Witwer and Lecavalier 2008; Sanders 2009; Woodbury-Smith and Volkmar 2009; Rinehart et al. 2010; Spek et al. 2010; Speirs et al. 2011; Nayate et al. 2012; Planche and Lemonnier 2012; Sharma et al. 2012; Lai et al. 2013; Tsai and Ghaziuddin 2014; Volkmar and McPartland 2014; Wilson et al. 2014; Barahona-Corréa and Filipe 2016; Montgomery et al. 2016; Prior et al. 1998).

According to Hans Asperger original description, his patients differ from those described by Kanner (Asperger 1944). Instead, Lorna Wing, translating Asperger’s work, named the syndrome and Kanner’s autism both part of an autistic continuum (Wing 1981).

AS definition and in its boundaries with HFA have been the topic of a growing body of literature, published from the 1980s till today, providing contradictory results: most researches have highlighted the significant similarities between AS and HFA (Howlin 2003; Macintosh and Dissanayake 2004; Witwer and Lecavalier 2008; Sanders 2009; Nayate et al. 2012; Wilson et al. 2014), while other authors continued to stress the importance to consider them as different clinical entities (Gilchrist et al. 2001; Ghaziuddin and Mountain-Kimchi 2004; Bennett et al. 2008; Ghaziuddin 2008; Speirs et al. 2011; Planche and Lemonnier 2012; Lai et al. 2013; Tsai and Ghaziuddin 2014; Barahona-Corréa and Filipe 2016; Montgomery et al. 2016).

Although a substantial overlap between AS and autism criteria does exist, a review of 69 studies showed that some fine differences between the two disorders can be recognized in terms of social interaction, motor skills and speech patterns; moreover, all these aspects seem to be relevant for designing clinical and intervention strategies (Sharma et al. 2012).

Tsai and Ghaziuddin (2014) examined 125 comparative studies between AS and HFA (autistic disorder and PDD-NOS); 30 studies concluded that the two conditions were similar, while 95 found quantitative and qualitative differences between them.

Taken together, the main evidences underline more quantitative rather than qualitative differences between AS to HFA, most of them regarding superior linguistic, cognitive and social functioning (Sanders 2009; Volkmar and McPartland 2014).

Recent studies largely focused on biological markers producing controversial results. Some of them found little support for a discrimination between AS and HFA, both in genetic and neuroimaging fields (Barahona-Corréa and Filipe 2016). Other studies underlined qualitative differences in the grey matter distribution between AS and HFA; these findings not only support the hypothesis that the two disorders might have different neurobiological basis, but also the evidence that mixing individuals with autism and AS may sometimes obscure important characteristics of one or the other condition alone (Yu et al. 2011; Bi et al. 2018).

### Asperger Syndrome and High Functioning Autism in the Age of DSM-5

Ultimately, the unsolved confusion in defining AS criteria and the clinical overlap between HFA and AS led to its merging into one unifying category, on the assumption that they cannot be reliably differentiated from one another; the latest edition of the DSM, the fifth, incorporated AS into Autistic Spectrum Disorder (ASD), removing the previously discrete diagnostic presentation of PDD (APA 2013). Furthermore, in order to better characterize the diagnosis, specifiers about the presence of cognitive and language impairment and severity levels have been added.

Multiple studies raised concerns about the number of individuals diagnosed with PDD (according to DSM-IV-TR) who may no longer match the new diagnostic criteria of DSM-5. Available literature shows contradictory results on this topic; in fact, some studies indicate that between 50 and 75% of individuals with a prior DSM-IV diagnosis of PDD will maintain the diagnosis, finding the greatest decreases among high functioning population (previous diagnosis of PDD-NOS or AS) (Kulage et al. 2014; Young and Rodi 2014; Smith et al. 2015). Other studies reveal similar percentage of HFA maintaining diagnosis (63%) and higher percentage only for AS (92%), concluding the absence of support for the concerns of losing the diagnosis with the advent of DSM-5 (Kim et al. 2015).

Another debated question is if the term of AS should be mentioned in the future version of the DSM as a “useful” label for a specific group of patients within ASD who differ in terms of clinical features, prognosis and treatment needs.

### Aims

The present study re-evaluated and compared subjects diagnosed according to DSM-IV-TR criteria with Asperger Syndrome (henceforth named AS group) and subjects diagnosed with PDD-NOS or AD with an IQ of average or above (henceforth named HFA group), and it aimed to: examine whether subjects with AS versus subjects with HFA differ in clinical profiles; evaluate the effect of the application of DSM-5 criteria and severity levels in terms of concordance between DSM-IV-TR and DSM-5; reflect about the utility and the clinical reliability of the merging subtypes of PDD in the broader diagnostic category of ASD.

## Methods

### Participants

Two groups of patients were sampled for the present study: a group with a diagnosis of AS (n = 80; age range 5–18); a group with HFA (n = 70, age range 3–18, including 7 patients with a diagnosis of Autistic Disorder and 63 patients with a diagnosis of PDD-Not Otherwise Specified).

Participants were recruited from a consecutive case series of children and adolescents (aged 3–18) assessed at the Neuropsychiatric Child Unit of the University of Bari, over the course of a 2-year period (2012–2013). Inclusion criteria: a clinical diagnosis of PDD, according to DSM-IV-TR criteria (APA 2000); intelligent quotient (IQ) average or above.

The total number of families invited to participate was 165 and 150 (90.9%) gave informed consent to participate.

### Materials

*Australian Scale for Asperger’s Syndrome* (ASAS) (Attwood 2006) is a questionnaire designed to identify behaviors and abilities indicative of AS in children, divided in five domains (social emotional abilities, communication skills, cognitive skills, specific interests, movement skills). Each domain consists of different questions, ranging from one (if the indicative behavior is rarely present) to six (if the indicative behavior is frequently present). Additionally, ASAS comprises a section of “other characteristics” that investigates the presence/absence of atypical sensitivity, stereotypic movements and language delay. ASAS results do not yield cut-off scores but give important information to be used in a clinical assessment. To provide a detailed account of the clinical profile of the subjects, we have chosen some specific items of ASAS as representative measures of clinical presentation. Responses to each question are in the form of “yes” (positive score) if the behavior is present (ASAS’s score 4–6), or “not” (not scored) if it is not (ASAS’s score 0–3).

Michigan Autism Spectrum Questionnaire (MASQ) (Ghaziuddin and Welch et al. 2013) is a scale designed on the basis of the clinical characteristics of HFA and AS. It focuses on two main areas: quality of social interaction and form/content of communication. It includes 10 questions, each has four responses ranging from 0 to 3, yielding a total score of 30. The highest total scores (> 22) predict AS, the intermediate scores (14 through 21) predict HFA and the lowest scores (< 14) predict other psychiatric disorders.

Intellectual functioning evaluation included the Wechsler Scales (WISC-IV in 69 patients, WISC-III in 49 patients, WPPSI-III in 32 patients); academic achievement of school-aged individuals (n = 117) has been evaluated with a battery of tests validated for the Italian language (Cornoldi and Colpo 1995, 1998; Sartori et al. 1995; Cornoldi et al. 2002, 2010; Cornoldi and Cazzola 2003); comorbidities assessment included the Italian version of the Child Behavior Checklist questionnaire (Achenbach and Edelbrock 1983; Achenbach 1991).

### Procedures

In the first step of the study, research team reviewed all the medical records of the participants, obtaining information about diagnoses and previous assessment (intellectual functioning, academic achievement and comorbidities). The research team was composed by one Child Psychologist and five Neuro-Psychiatrists.

In the second step of the study, research team administered to caregivers two rating scales designed to measure specific core features of AS and HFA: the Australian Scale for Asperger’s Syndrome (Attwood 2006) and the Michigan Autism Spectrum Questionnaire (Ghaziuddin and Welch 2013).

In the last step of the study a comparison of the two groups in term of cognitive functioning, core clinical features, school learning abilities and comorbidities was conducted. Moreover, an analysis of the consequences of the application of DSM-5 criteria in terms of concordance and severity levels was performed.

### Statistical Analysis

Demographic data, including age and sex, between the two groups, underwent statistical analysis (Table 1).

**Table 1:** Demographic characteristics of the sample

	AS		HFA		Test	df	p value
	n	%	n	%			
Sex							
M	66	82.5	61	87.1	0.31384*	1	0.5753
F	14	17.5	9	12.9			
Age							
Mean (SD)	11.5 (3.8)		8.9 (3.8)		4.16^#^	148	< 0.0001
Range	5–18		3–18				

*SD* standard deviation, *AS* Asperger Syndrome, *HFA* high functioning autism, *df* degree of freedom

*Test Chi square

^#^Test* t* student for independent groups

Quantitative data were summarized as mean and standard deviation. Because quantitative data resulted normally distributed, comparisons between independent groups have been performed with* t* student test. Multiple evaluations of scales were compared between AS and HFA groups with a multiple testing approach, using a* t* test and adjusting p values with a permutational false discovery rate (FDR) method.

Analysis of variance for repeated measure was applied to evaluate difference between AS and HFA groups in two score methods (paired measure).

Qualitative variables are summarized as count and percentage, comparisons among two or more group were performed with Chi square test or Fisher exact test, as appropriate. Comparisons of percentage of answers to questionnaires between AS and HFA groups were performed by a multiple testing approach with a permutational FDR adjustment of p values. Difference in percentage and its 95% confidence interval was determined for each single comparison accounting the binomial method.

Concordance between DSM-IV (AS and HFA) and DSM-5 (level 1, level 2, level 3) was evaluated with McNemar test, the strength of the concordance was described by Cohen’s K.

To evaluate the discriminating power of MASQ a ROC curve analysis was performed, reporting area under the curve (AUC) and its 95% confidence interval.

Main comparisons were considered as statistically significant for p value < 0.05. Analysis was performed with SAS 9.4 for PC and R version 3.2.3. The PROC MULTTEST was applied to account for multiple testing, using the permutational FDR approach. ROC curve analysis was conducted with MedCalc Statistical Software version 18 (MedCalc Software bvba, Ostend, Belgium; http://www.medcalc.org; 2018).

## Results

### Intelligence Quotient (Table 2)

**Table 2:** Intelligence quotient from Weschler scales

	AS	HFA	Raw p value	Adjusted p value	Difference AS-HFA (95% CI)	*t* test (df)
	Mean (SD)	Mean (SD)				
Full scale intelligent quotient (FSIQ)	114.1 (15.9)	92.1 (14.5)	< 0.0001	< 0.0001	22.04 (17.08–26.99)	8.78 (147)
WISC-IV						
Verbal comprehension index (VCI)	122.0 (16.4)	96.1 (18.8)	< 0.0001	< 0.0001	25.94 (17.19–34.69)	5.92 (66)
Perceptual reasoning index (PRI)	117.5 (14.8)	93.5 (13.7)	< 0.0001	0.0002	23.95 (16.67–31.23)	6.57 (67)
Working memory index (WMI)	103 (17.4)	84.5 (14.8)	< 0.0001	< 0.0001	18.56 (10.1–27.03)	4.38 (64)
Processing speed index (PSI)	94.3 (17.1)	82.5 (10.9)	0.0017	0.0017	11.77 (4.55–18.98)	3.25 (77)
WISC-III						
Verbal intelligent quotient (VIQ)	112.7 (16.1)	93.4 (16.4)	< 0.0001	< 0.0001	19.31 (10.47–28.14)	4.38 (54)
Performance intelligent quotient (PIQ)	109.2 (15.7)	91.9 (16.7)	0.0002	< 0.0001	17.26 (8.68–25.84)	4.03 (56)

*AS* Asperger syndrome, *HFA* high functioning autism, *CI* confidence interval, *df* degree of freedom

The mean (SD) of FSIQ was 114.1(15.9) in AS and 92.1(14.5) in HFA, showing a statistically significant higher value in the former group (*t* test = 8.78, raw p value < 0.0001, adjusted p value < 0.0001). Moreover, all the IQ sub-values resulted statistically higher in AS than in HFA (Table 2).

### Core Clinical Features, Obtained from ASAS Scores (Table 3)

**Table 3:** Core clinical features obtained from ASAS

ASAS domains	AS (n = 80)		HFA (n = 70)		Differences AS-HFA (95% CI)	Raw p value	Adjusted p value
	n	%	n	%			
Social emotional abilities							
Unawareness of the unwritten rules of social play	48	60.0	48	68.6	− 8.5% (− 25.2 to 8%)	0.309	0.997
Avoid social contact	29	36.3	35	50.0	− 13.7% (− 30.8 to 3.3%)	0.1	0.781
Excessive amount of reassurance	60	75.0	59	84.3	− 9.3% (− 23.4 to 4.8%)	0.225	0.972
Not interested in competitive sports and activities	28	35.0	30	42.9	− 7.9% (− 24.8 to 9.1%)	0.401	1
Communication skills							
Literal interpretation of comments	23	28.8	35	50.0	− 19% (− 35.9 to − 2.1%)	0.0114	0.0431
Unusual tone of voice	57	71.3	47	67.1	4.2% (− 12.1 to 20.5%)	0.5995	1
Uninterested in other side of the conversation	56	70.0	43	61.4	9.1% (− 7.5 to 25.7%)	0.3024	0.996
Less eye contact when in a conversation	40	50.0	42	60.0	− 11.2% (− 28.6 to 6.2%)	0.2516	0.988
Over-precise or pedantic speech	45	56.3	21	30.0	32.2% (3.6–38.9%)	0.0017	0.0112
Cognitive skills							
Exceptional long-term memory for events/facts	58	72.5	37	52.9	10.5% (− 6.9 to 28%)	0.0172	0.0511
Specific interests							
Fascinated by a particular topic	63	78.8	47	67.1	12.9% (− 3.4 to 29.2%)	0.1389	0.876
To be unduly upset by changes in routine	53	66.3	49	70.0	− 3.2% (− 20.4 to 14.1%)	0.7261	1
To develop routines/rituals	40	50.0	38	54.3	− 6.3% (− 24.7 to 12%)	0.6263	1
Movement skills							
Poor motor coordination	50	62.5	44	62.9	− 5.2% (− 22.4 to 12.1%)	1	1
Other characteristics							
Unusual fear/distress due to light touch on skin or scalp	25	31.3	25	35.7	− 4.7% (− 21.8 to 12.3%)	0.605	1
Unusual fear/distress due to unexpected noises	45	56.3	32	45.7	5.7% (− 12.9 to 24.5%)	0.2518	0.99
A tendency to flap or rock when excited/distressed	38	47.5	39	55.7	− 6.8% (− 24.8 to 11.3%)	0.3308	1
Late in acquiring speech	26	32.5	39	55.7	− 23.2% (− 40.1 to − 6.4%)	0.0051	0.0234

*AS* Asperger syndrome, *HFA* high functioning autism, *CI* confidence interval

Among social emotional abilities, there was no significant difference between the two groups, nevertheless HFA appeared more compromised than AS subjects, especially in avoiding social contact.

Among communication skills, over-precise or pedantic speech was significantly more common in AS than HFA subjects (raw p value = 0.0122, adjusted p value = 0.0112); on the contrary literal interpretation was significantly more common in HFA than AS subjects (raw p value = 0.0114, adjusted p value = 0.0431).

Among cognitive skills, there was no significant difference between the two groups, though exceptional long-term memory and fascination by a particular topic was more common in AS group.

No statistically significant difference in clumsiness (both raw and adjusted p value = 1), atypical sensitivity (raw p value = 0.605; p value = 1) and motor mannerism was observed between the two groups.

Delay in acquiring speech was significantly more common in HFA compared to AS group (raw p value = 0.0051, adjusted p value = 0.0234).

### MASQ Scores (Fig. 1)

**Fig. 1:**
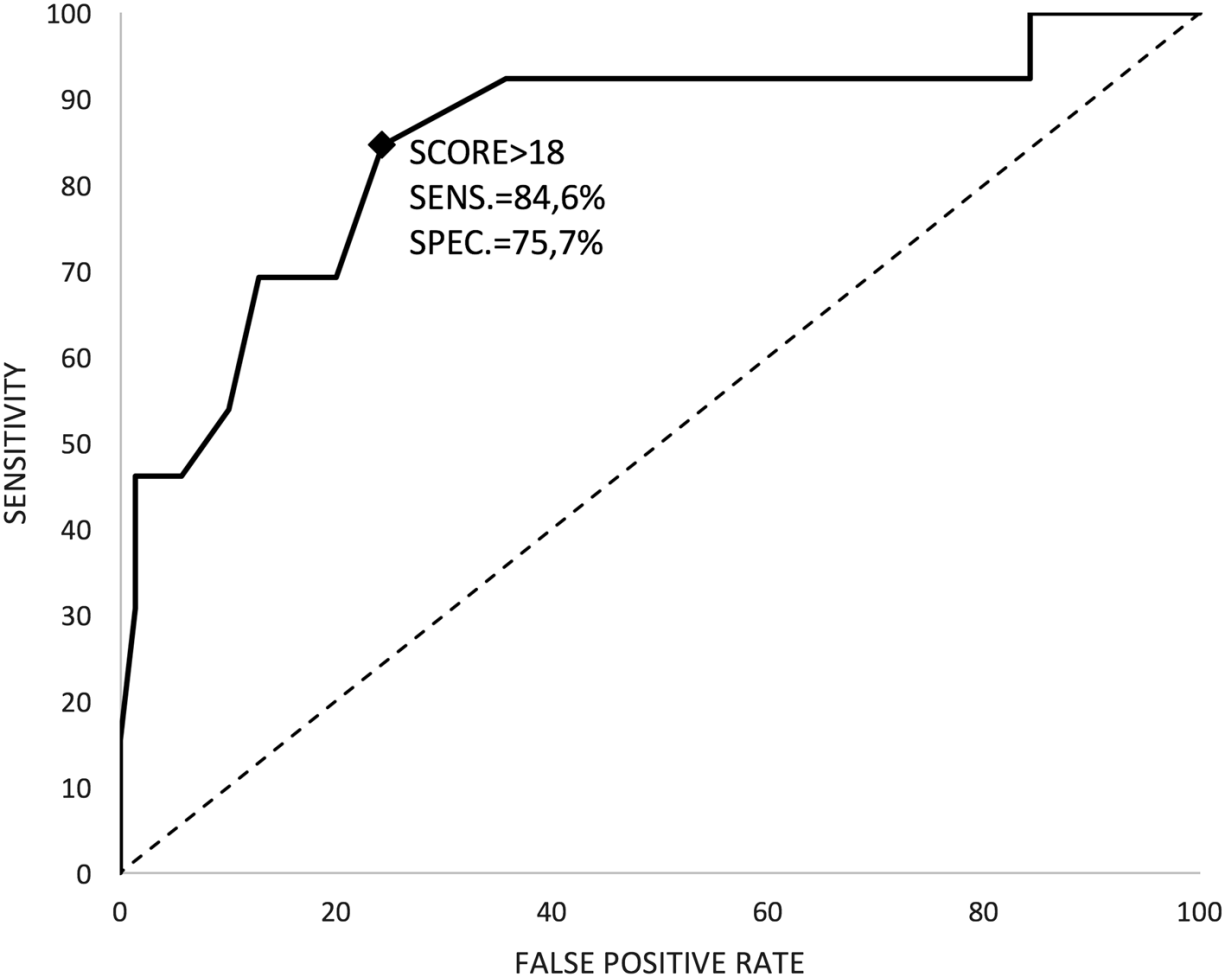
ROC curve for AS versus HFA. *AS* Asperger syndrome, *HFA* high functioning autism

The mean (SD) score was 22.2 (5.1) in AS group and 15.5 (5.9) in HFA group, showing a statistically significant higher value in the former group (*t* test = 7.42, p value < 0.0001). In the ROC analysis, AUC resulted 0.85 (95% CI 0.72–0.98). The suggested threshold is a score of 18 that provides a sensitivity of 84.6% (95% CI 54.6–98.1) and specificity of 75.7% (95% CI 64–85.2).

### School Learning Abilities (Table 4)

**Table 4:** Low performance in school learning abilities

	AS		HFA		Raw p value	Adjusted p value	Difference AS-HFA (95% CI)
	(n = 69)		(n = 48)				
	n	%	n	%			
Reading (time)	14	20.3	20	41.7	0.0142	0.015	− 21.4% (− 40.01 to − 2.74%)
Reading (accuracy)	12	17.4	17	35.4	0.0320	0.0299	− 18% (− 34.2 to − 0.04%)
Reading (comprehension)	12	17.4	20	41.7	0.0058	0.0074	− 24.3% (− 42.6 to − 5.94%)
Mathematics (time)	23	33.3	25	52	0.0562	0.0431	− 18.7% (− 38.5 to 1%)
Mathematics (accuracy)	16	23.2	25	52.1	0.0013	0.0033	− 28.9% (− 47.9 to − 9.8%)
Writing (accuracy)	27	39.1	34	70.8	0.0017	0.0033	− 31.7% (− 50.7 to − 12.7%)
Handwriting	42	60.8	35	72.9	0.2347	0.1754	− 12.1% (− 30.9 to 6.8%)

*AS* Asperger syndrome, *HFA* high functioning autism, *CI* confidence interval

Analyzing the subsample of 117 school-aged subjects, learning difficulties were significantly more frequent in HFA than in AS. In particular, the frequency of impairment in reading (adjusted p values: time, p value = 0.015; accuracy, p value = 0.0299; comprehension, p value = 0.0074), mathematics (time, p value = 0.0431; accuracy, p value = 0.0033) and writing (accuracy, p value = 0.0033) was significantly higher in HFA respect to AS group; only the handwriting impairment was not significantly different between the two groups (p value = 0.1754).

### Comorbidities (Table 5)

**Table 5:** Comorbidities

	AS		HFA		Test	p value	Difference AS-HFA (95% CI)
	n	%	n	%			
At least one comorbidity	59	49.1	33	42.9	11.14 (1)	0.0013	6.2% (10.11 to 43.1%)
ADHD	17	21.2	19	27.1	0.7108 (1)	0.3992	− 5.9% (− 20.9 to 9.19%)
Depressive disorder	10	12.5	2	2.9	4.7166 (1)	0.0299	9.6% (0.07 to 19.2%)
Bipolar disorder	5	6.2	0	0		0.0611*	6.2% (− 0.4 to 12.9%)
Obsessive–compulsive disorder	4	5	2	2.8		0.6854*	2.2% (− 5.4 to 9.6%)
Eating disorder	2	2.5	1	1.4		1*	1.1% (− 4.4 to 6.55%)
Anxiety disorder	25	31.2	3	4.3	17.8788 (1)	< 0.0001	16.9% (14.4 to 39.5%)
Tic disorder	6	7.5	2	2.9		0.2847*	4.6% (− 3.66 to 12.9%)

*AS* Asperger syndrome, *HFA* high functioning autism, *CI* confidence interval

*p values from Fisher Exact test and exact 95% confidence interval for the difference of proportions

The presence of at least one comorbidity was significantly higher in AS than HFA group (Chi square = 11.14; p value = 0.0013). In both groups, the most frequent comorbidity was ADHD, but comparing AS and HFA, its prevalence did not statistically differ (p value = 0.3992). Depressive and anxiety disorders were significantly more common in AS than in HFA group (Chi square = 4.71; p value = 0.0299; Chi square = 17.87; p value < 0.0001); none of other comorbidities showed a statistically significant difference between the two groups.

### Application of DSM-5 Criteria and Severity Levels

When DSM-5 criteria for ASD were applied, these were met by the 97.3% of the total sample 100% of HFA (70/70) and 95% of AS (76/80).

Applying DSM-5 severity levels, 95% of AS subjects (76/80) were classified as Level 1 (the reminder 5% was not classified in ASD); 55.7% of HFA subjects were classified as Level 1 and 44.3% as Level 2. The concordance measured with Cohen’s K was 0.42 (95% CI 0.29–0.54, Mc Nemar statistic = 43, df = 3, p < 0.0001).

## Discussion

In DSM-5, the diagnostic label of Asperger’s syndrome (AS) was removed and it was included in a more general category of Autism Spectrum Disorder (ASD). So, its clinical validity, already rather debated, became more ambiguous and its differentiation from High Functioning Autism (HFA) remained unsolved or, at least, equivocal.

Irrespective of the DSM-5 changes, several studies (also published later than APA 2013) continue to debate about AS and its conceptualization within ASD (Chiang et al. 2014; Volkmar and McPartland 2014; Tsai and Ghaziuddin 2014; Wilson et al. 2014; Lai et al. 2015; Helles et al. 2015; Montgomery et al. 2016; Faridi and Khosrowabadi 2017). In this unclear perspective, this study provided a detailed description of clinical profiles of 150 AS/HFA subjects, aged 3–18 years, to examine whether it has been useful to merge them in a unique diagnostic category or if it would be more meaningful to differentiate them in distinct diagnostic categories.

### Asperger Syndrome Versus High Functioning Autism

First, in the present study, the results suggest that in both AS and HFA, autistic core features (i.e. deficits in social communication and social interaction and restricted, repetitive patterns of behavior, interests, or activities) are present, even if with subtle differences.

### Communication

About the communication features, the present study revealed a significant difference in the presence of a history of delay in language development, prior to 3 years of age, between subjects with AS and subjects with HFA, even if also in AS a mild language delay was reported. In the DSM-IV-TR, the “absence of clinically significant general delay in language (e.g., single words used by age 2 years, communicative phrases used by age 3 years)” was a relevant criterion for AS diagnosis. In contrast with this theoretical construct, extensive literature data showed that language delay could not be considered as a diagnostic exclusion criterion for AS because subtle language problems are expected in AS and also because the recognition of a language delay is not always enough reliable, since it is often dependent on parents recall (Ghaziuddin and Welch 2013). In the DSM-5, the decision to merge the single diagnostic entities in a unique broader category of ASD and the introduction of language impairment as a “specifier” solved this controversy. The present study suggests that even if language delay may not be a differentiating feature of AS/HFA, it can be useful to differentiate developmental language profiles in terms of clinical approach. In the present study, communication skills resulted overall overlapping between AS and HFA, revealing statistically significant differences only in “over-precise or pedantic speech” (characterized by overly formal speech, similar to an in-depth monologue about a topic of special interest, verbose and tangential, lack of the normal prosody), more frequent in AS, and in “literal interpretation of comments”, more common in HFA. The first finding (“over-precise or pedantic speech”) is unsurprising and represents one of the most typical clinical feature of AS, firstly described by Hans Asperger’s original work (1944, in which AS were called “little professors” for their way of talking), then included in the following proposed diagnostic criteria sets (Gillberg and Gillberg 1989; Szatmari et al. 1989) and finally confirmed by subsequent studies (Ghaziuddin and Gerstein 1996; Gilchrist et al. 2001; Macintosh and Dissanayake 2004; Woodbury-Smith and Volkmar 2009). The second finding (“literal interpretation of comments”) may be related to the HFA worse cognitive and comprehension abilities, which made them unable in recognizing the speaker’s communicative intention and in social understanding, unlike patients with AS, who adopted intellectual strategies of compensation.

### Motor Functions

Clumsiness has been originally considered a distinctive feature of AS (Ghaziuddin et al. 1994); in our experience, it appeared in both groups, without significant difference, as reported by more recent studies (Fournier et al. 2010; Nayate et al. 2012).

### Sensory System

Even if sensory abnormalities were well-represented in our sample, no significant difference between subjects with AS and subjects with HFA was observed. Sensory abnormalities were included in DSM-5 within the second cluster of core symptoms (previously omitted). Sensory overload can be experienced as painful and correlated with lower participation in leisure activities and lower performance at school, therefore considering sensory features in intervention is essential (Grapel et al. 2015).

### Intellectual Functioning

In this study, Full Scale Intelligence Quotient mean values were statistically significant higher in AS than HFA. This result is in accordance with previous literature data (Ghaziuddin and Mountain-Kimchi 2004; Macintosh and Dissanayake 2004; Koyama et al. 2007; Ghaziuddin 2008; Witwer and Lecavalier 2008; Noterdaeme et al. 2010; Chiang et al. 2014), although, on this topic, conflicting results were reported. Wilson et al. compared cognitive characteristics of individuals with AS and HFA reporting no significant differences on the cognitive performance (2014). Most reports suggested that patients with AS have a distinct cognitive profile, characterized by a higher verbal IQ and a lower performance IQ, whereas in most cases with HFA, the pattern is reversed (Ghaziuddin and Gerstein 1996; Saulnier and Kin 2007; Volkmar et al. 2009; Noterdaeme et al. 2010; Planche and Lemonnier 2012; Chiang et al. 2014). This profile has been linked to the better verbal abilities of AS patients. Other studies did not confirm the existence of these typical profiles, reporting mixed cognitive patterns that are not consistent enough to enable diagnostic differentiation between the two groups (Ghaziuddin and Mountain-Kimchi 2004; Macintosh and Dissanayake 2004; Ghaziuddin 2008). In the present study, on the basis of the cognitive evaluation, it is not possible to distinguish the two groups: all the IQ sub-values resulted statistically higher in AS than in HFA and differences between verbal and performance sub-values are not statistically significant within the two groups. These results support the hypothesis that the two conditions could be distinguished at a quantitative cognitive level, represented by FSIQ, rather than at a qualitative level, represented by specific cognitive profile. Nevertheless, as for intellectual disability, the presence of very different IQs could denote distinct levels of severity and clinical profile, in the same way, a large discrepancy between AS and HFA intellectual scores could have a qualitative effect on general function.

### Academic Achievement

In the present study, another remarkable topic of differentiation between HFA and AS is the prevalence of learning disorders that resulted significantly more frequent in HFA, except to dysgraphia that emerged frequently in both groups, without a significant difference. Low performances in hand-writing, in terms of forming letters or using the paper space were noted in the original description of AS (Asperger 1944) and in recent literature (Jansiewicz et al. 2006; Fuentes et al. 2009). To our knowledge, until today, academic functioning and education needs in HFA have still not been sufficiently explored in literature. In accordance with our findings, Grimm et al. (2015) reported schooling of 45 patients exhibiting ASD without mental retardation, finding that patients with childhood autism and atypical autism respect to those with AS were more likely to receive the support of a special educational assistant and underwent a higher number of consultations and treatment episodes.

### Comorbidities

Rates of comorbid disorders are significantly higher in AS than HFA. The most prevalent comorbidity in both groups is ADHD, but comparing AS and HFA, its prevalence did not statistically differ. An association between ASD and ADHD and other externalizing disorders have been reported and supported by clinical population research and twin studies, suggesting substantial genetic overlap (Taylor et al. 2013, 2015; Cooper et al. 2014; Salazar et al. 2015; Antshel et al. 2016). Considering that comorbidity between these disorders was a relevant and frequent occurrence, differently from the previous edition, DSM-5 allowed the diagnosis of ADHD in the context of an ASD. People with AS displayed more anxiety and depressive symptoms compared to HFA, consistently with previous literature (Mazzone et al. 2012; Simonoff et al. 2013; Ung et al. 2013; Chiang and Gau 2015; De-la-Iglesia and Olivar 2015; Roy et al. 2015; Gillberg et al. 2016).

### Clinical and Treatment Implications

Overall, the results of this study suggested a complex aged-related different developmental profile between HFA and AS, characterized by quantitative and qualitative differences such as language development and communication style, academic achievement, comorbidities profile and cognitive functioning. Since language development and academic abilities play a central role in global functioning during the first years of life, HFA require more support in terms of rehabilitative treatments and school educational needs than AS. Consistently, in this study, when DSM-5 specifiers of severity levels were applied to the sample, HFA were more frequently classified in level 2 (“requiring substantial support”) than AS, while AS were more frequently classified in level 1 (“requiring support”) than HFA.

Nevertheless, despite this aged-related definite pattern of differentiation and severity level classification, in middle childhood and adolescence, AS presents higher internalizing comorbidities rate (overall anxiety and depressive disorders) than HFA, as resulted in this study. The presence of high rate of comorbidity is also underlined in previous literature (Mazzone et al. 2012; Simonoff et al. 2013; Ung et al. 2013; Roy et al. 2015; Gillberg et al. 2016). This could be explained with AS higher cognitive and communicative levels, that made them more able in introspection, more “active but odd” (Ghaziuddin 2008; Wing et al. 2011) in relations attempts, more conscious of social difficulties and so more vulnerable to affective disorders (De-la-Iglesias and Olivar 2015).

### Current and Proposed Specifier

At present, DSM-5 allows to use specifiers of language impairment, intellectual impairment and severity levels to distinguish clinical profiles of ASD broader category. Nevertheless, considering the results obtained from the clinical profiles comparison and from the DSM-5 severity levels distinction, it is necessary to consider the risk that, in the severity specifier “Level 1” of the DSM-5, many phenotypic variances could be squeezed making this level very heterogeneous. Because of this, any attempts to predict clinical outcomes, develop individualized treatment targets or identify etiological factors may become unreal (Kim et al. 2015). Lai et al. (2013) underlined that defining autism using the umbrella term ASD, could hide the evident heterogeneity. In order to make progress in autism research and ultimately improve clinical practice, there is the necessity to move forward in the identification of subtypes within the autism spectrum. Maybe, a “subtype specifier” for HFA and AS could be useful to direct specific age-related paths of treatment (i.e. services of traditional rehabilitation, such as speech therapy and psychomotricity, and of assistance tools for school targeted to HFA and social training and psychotherapy targeted to AS).

Clinical judgement of qualified professional experts and the use of specific scales, designed on the clinical characteristics of HFA and AS, could provide data for this subtle distinction. In the present study, MASQ gave significantly different scores in HFA and AS, so that its application could help in discriminating AS from HFA subjects. In its original description, MASQ was used to distinguish, beyond AS and autism, high functioning spectrum disorders from other psychiatric disorders. Authors found a threshold of 22 for AS and intermediate scores for autism/PDD NOS (Ghaziuddin and Welch 2013).

In the present study, the suggested threshold is lower (18 with a sensitivity of 84.6% and specificity of 75.7%).

### Concordance Between DSM-IV-TR and DSM-5

The second aim of this study was to verify the percentage of individuals maintaining diagnoses when DSM-5 diagnostic criteria were applied. Several reviewed studies found that DSM-5 criteria for ASD have a higher specificity but lower sensitivity as compared to the DSM-IV-TR (Gibbs et al. 2012; Worley and Matson 2012; Koh et al. 2014; McPartland et al. 2014). Recent studies indicated a percentage between 50 and 75% of individuals maintaining diagnosis under DSM-5 criteria, with the greatest decreases among high-functioning populations or previous diagnoses of PDD-NOS or AS (Kulage et al. 2014; Young and Rodi 2014; Smith et al. 2015). In the present study, 97.3% of the total sample met DSM-5 criteria for ASD; the remainder, who did not meet the full criteria, consisted exclusively of AS subjects. These AS subject, even if in presence of cognitive-emotional-social features representative of AS, lacked, at the age of re-examination, of significant clinical global impairment, indispensable to justify the diagnosis. In fact, recent theories of neurodiversity that consider AS a normal human difference rather than a disorder (Baron-Cohen 2002; Jaarsma and Welin 2012; Lorenz and Heinitz 2014), their phenotypes may be considered as a sub-clinical AS forms. Nevertheless, also approving the hypothesis of sub-clinical phenotype, in most cases, these subjects also need a sort of support (cognitive-behavioral treatment, parent/teacher psycho-education, personalization of school program). As in ADHD coding of DSM-5, regarding level 1 ASD patients, it could be suggested the use of a “partial remission” specifier, for those patients who do not meet currently the full diagnostic criteria.

As mentioned above, in the present study, the severity levels application showed a distribution of HFA in level 1 and 2, while AS were totally placed in level 1. The concordance between HFA/AS groups and level 1/level 2 groups, measured with Cohen’s K, could be considered poor. Indeed, the choice of evaluating the concordance using McNemar test was forced, since the matched classifications were not concordant: HFA/AS groups pertained to a clinical perspective, while level1/level 2 to an assistential one. In daily practice, clinicians do not strictly address type and intensity of support only to diagnoses, but they have to consider lots of variables, depending on age, comorbidities and environment.

## Conclusion

Considering the available evidences and our results, even continuing to use the broad category of ASD, as Wing (who firstly rescued Asperger’s work) stated, “a number of changes need to be made in order that the future version of DSM might be used reliably and validly in clinical practice and research” (Wing et al. 2011). ASD category of DSM-5 had the merit to have solved lots of previous classification discordances and “splitting” approaches (Volkmar and McPartland 2014). Nevertheless, it could be meaningful to introduce an additional “subtype specifiers” (i.e., Autistic Disorder or Asperger’s disorders), in order to grant the recognition of different “nuances” of the spectrum and determine better individual clinical, assistential and research addresses.

### Limitations and Future Directions

The sample of the present study is characterized by a preponderance in HFA group of PDD-NOS that tend to be rather heterogeneous. In previous research, comparisons were made with samples that include larger number of AD. Therefore, it is unknown whether our results may be extended to the broader ASD category. Lastly, in the evaluation of academic achievement, the higher rates of learning problems in children with HFA might be attributed or at least influenced by the lower IQ scores of HFA than AS. Future research about the efficacy of these prospective specifiers could be beneficial.
